# Interactions between soil and other environmental variables modulate forest expansion and ecotone dynamics in humid savannas of Central Africa

**DOI:** 10.1098/rspb.2024.1120

**Published:** 2024-10-30

**Authors:** Le Bienfaiteur Takougoum Sagang, Imma Tcheferi, Pierre Ploton, Moses Libalah, Murielle Simo-Droissart, Nelly Sirri, Gilles Dauby, Eric Ngansop, Jean Pierre Bissek, Narcisse Kamdem, Gislain I. I. Mofack, Donatien Zebaze, Hugo Leblanc, Fabrice Djonko, Bonaventure Sonké, Nicolas Barbier, Pierre Couteron

**Affiliations:** ^1^ Institute of the Environment and Sustainability, University of California, Los Angeles, CA 90095, USA; ^2^ Department of Biology, Plant Systematics and Ecology Laboratory, Higher Teachers’ Training College, University of Yaoundé I, P.O. Box 047, Yaoundé, Cameroon; ^3^ IRAD-Njombé Research Station, P.O. Box 13, Njombé, Cameroon; ^4^ AMAP, Univ Montpellier, IRD, CNRS, INRAE, CIRAD, Montpellier 34394, France; ^5^ Department of Plant Biology, Faculty of Science, University of Yaoundé I, P.O. Box 812, Yaoundé, Cameroon; ^6^ IRAD-National Herbarium of Cameroun, P.O. Box 1601, Yaoundé, Cameroon; ^7^ Ministry of Forestry and Wildlife, Mpem & Djim National Parc, P.O. Box 05, Ntui, Cameroon; ^8^ Gembloux Agro- Bio Tech, TERRA Teaching and Research Centre, Forest is Life, University of Liège, Gembloux, Belgium; ^9^ International Joint Laboratory DYCOFAC, IRD-UYI-IRGM, P.O Box 1857, Yaoundé, Cameroon

**Keywords:** alternative ecosystem states, Central Africa, forest expansion, grass–fire feedback, savanna community, soil

## Abstract

Forest expansion into savanna is a pervasive phenomenon in West and Central Africa, warranting comparative studies under diverse environmental conditions. We collected vegetation data from the woody and grassy components within 73 plots of 0.16 ha distributed along a successional gradient from humid savanna to forest in Central Africa. We associated spatially collocated edaphic parameters and fire frequency derived from remote sensing to investigate their combined influence on the vegetation. Soil texture was more influential in shaping savanna structure and species distribution than soil fertility, with clay-rich soils promoting higher grass productivity and fire frequency. Savanna featuring woody aboveground biomass surpassing 40 Mg ha^−1^ could escape the grass–fire feedback loop, by depressing grass biomass below 4 Mg ha^−1^. This thicker woody layer also favoured the establishment of fire-tolerant forest pioneers, which synergically contributed to the expansion of forests. Conversely, savannas below this fire suppression threshold sustained a balance between trees and grasses through the grass–fire feedback mechanism. This hysteresis loop, particularly pronounced on clayey soils, suggests that the contrast between grassy savanna and young forests might represent alternative ecosystem states, although savannas with low woody biomass remained vulnerable to forest edge encroachment.

## 1. Introduction

Woody encroachment and forest expansion into tropical savanna have been described as top drivers of biome transition [1]. In Central Africa, the interface between forest and savanna is the most dominant ecotone [2,3], where sizable tracts of savannas are juxtaposed with close canopy forest resulting in mosaic landscapes [3–6]. This situation has led some authors interpreting humid savannas and forests as alternative ecosystem states (AES) under the currently prevailing conditions [4–8]. Indeed, these regions have mean annual precipitation (MAP) exceeding 1400 mm year^−1^, which can support closed-canopy forests [9,10] and has favoured widespread forest expansion into savanna over recent decades [11–15]. Even though neither mosaic landscapes nor forest expansion are unambiguous clues of AES [8], the forest–savanna transition fringing the Congo Basin has been mapped as possible place of AES occurrence by most authors [6,14,16,17] and thereby deserves further scrutiny. Moreover, forest encroachment in these ‘zones of tension’ [18,19] comes with profound changes in function and composition of the initial savanna ecosystem [20–22]. This has generated considerable interest to ecologists as various key ecosystem functions (including functional diversity, nutrient cycling and hydrology), services (among which carbon sequestration) and production (livestock) depend on woody versus grassy proportions in a savanna [23–26].

The coexistence of forest and savanna under similar wet climates has triggered two hypotheses. The first one, compatible with AES existence refers to feedback between vegetation structure and disturbances, notably fires that prevent savannas from canopy closure by limiting the development of woody saplings [27–29]. Fire intensity and spread depend on the aboveground biomass (AGB) and flammability of a continuous heliophytic C_4_ grass layer [30–33]. Several studies have highlighted a level of grass AGB around which fire propagation swiftly increases (fire propagation threshold [30,31,33]). Within savanna landscapes, woody AGB has little influence on fire behaviour until the woody canopy cover becomes sufficient to strongly depress heliophytic flammable grasses below this fire propagation threshold. Furthermore, higher canopy can even eliminate fire from spreading within the understory (fire suppression threshold [7,34,35]). These two antagonistic feedback (i.e. grass–fire versus tree–shading [30,34,36–38]) are not only central to the ‘savanna conundrums’ (*sensu* [39]), but are also presented as source of possible vegetation switches between AES [4,7,35] (i.e. forests and open vegetation in the form of savanna or grassland) [40–42].

The second line of interpretation of forest–savanna mosaics refers to abiotic factors, notably those emanating from the underlaying substrate and deriving edaphic conditions. Some studies have emphasized spatial correlation between vegetation structure and soil variables (i.e. texture and/or nutrient availability) at landscape scale [17,43–46]. For example, extensive savanna tracts in the Bateke plateaus in Central Africa are found on poor edaphic conditions (low fertility) deriving from very sandy substrates [47–50], while adjacent geological substrates displaying soils of finer texture mainly harboured close canopy forests. Such observations have fostered caveats against interpreting contrasted forest–savanna mosaics as unequivocal evidence of AES and even led some authors to questioning the effectiveness of fire alone in stabilizing open ecosystem states as alternative to forest [32,51]. Both the ‘internal dynamics’ and ‘external forcing’ perspectives [8] concur that edaphic factors can interplay with fire disturbances to shape ecosystem characteristics. Understanding these interactions through vegetation metrics is crucial for enhancing resource management and conservation efforts in forest–savanna transition zones. The outcomes of interactions between savanna dynamics and soil properties are *a priori* uncertain and therefore deserve thorough investigation. On the one hand, productive soils are expected to enhance the growth of woody saplings and let them better benefit from fire return intervals to escape the fire trap, which further promotes canopy cover build-up [31,52]. But productive environments can also boost grass production [53,54] and are often associated with higher fire intensity and frequency [37,46,55–57]. These two simultaneous effects make non-trivial the influence of soil productivity gradients on the dynamical result of fire-mediated tree–grass interactions [46].

While most studies report forest-favouring dynamics, spatial analysis from remote sensing data shows that the progression of forest does not always consist of a regular advance [15–17,58,59]. Forest progression can be either fast, through the growth and coalescence of woody patches within savanna (also referred to as ‘nucleation’), which causes the edge to smoothen [16,55], or slow, through boundary expansion of existing forest [14,16]. Such observed spatial and temporal heterogeneity of forest encroachment modes likely overlap intrinsic site abiotic/biotic constraints liable to modulate the thresholds involved in the fire-mediated tree–grass feedback. Different studies [13,16,58,60,61] underlined the influence of the floristic composition of the ecotone and described a structurally and functionally distinct ecotonal tree community dominated by forest pioneers [58,59,62]. This ecotone was reported as spatially extensive and promoting woody encroachment by shading out grasses and attenuating the grass–fire feedback on trees. This systematic effect of the woody species composition of the ecotone is, however, challenged in some areas [63], where limited discontinuity in the floristic composition across a forest–savanna gradient is found.

Modelling is of course central to elucidate and disentangle such complex dynamics and substantial modelling efforts have logically stemmed from the savanna conundrum [64–66]. However, theoretical development in modelling has not yet been matched by sufficient empirical work considering the diversity of local biotic and abiotic conditions along with the variety of dynamics observed at forest–savanna interface. Notably, while the woody component of the savanna is quantified in different ways (stem counts, LAI, cover, etc. [39]), there are few existing sets of spatially collocated data of grass, trees and fire, making inter-study comparison and model calibration difficult. In addition, the locations of most of the studies describing African forest–savanna transition under MAP beyond 1200 mm (i.e. Lamto in Ivory Coast [67,68], Lopé in Gabon [13,69] or Kogyae in Ghana [63]) were found on oligotrophic sandy soils. Detailed field information on more productive edaphic conditions is therefore lacking.

The general objective of this study was to assess how the interaction between soil properties and other environmental variables modulates the grass–fire feedback and changes in woody structure and floristic composition amidst ongoing forest encroachment into savanna (which is overall swift but variable in space). We rely on a comprehensive dataset from spatially collocated remote sensing-based products and field measurements (73 field plots of 0.16 ha) collected in the forest–savanna mosaic of the Guineo–Congolian transitional area of Cameroon. Floristic composition of the woody layer, wood and grass AGB, together with the topsoil properties (<20 cm depth) were sampled within savanna, and recent colonizing forest (<10 years since forest gain) along a wide range of soil texture/fertility profiles. Data from Landsat-derived monitoring of fire frequency and forest age over 45 years (as per [15]) were associated to these data to address the following questions:

- What is the influence of soil texture and fertility on grass biomass productivity and to what extent does the variability in the later influences fire frequency?
- What are the limits of this grass–fire feedback control on tree development and associated shading effect on grasses?
- How do plant functional types (forest pioneers versus savanna-specialist) contribute to the structure and composition of the woody layer across a forest–savanna transition?
- How do soil and other environmental factors correlate with the main gradients of woody species composition?

## 2. Material and methods

### (a) Study site

**Figure 1.**
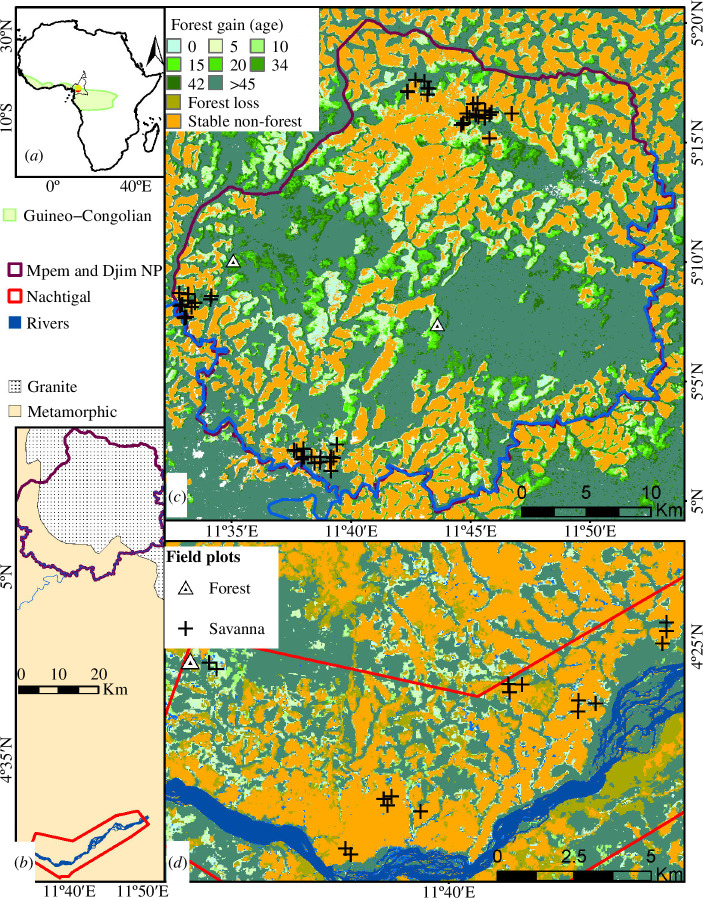
Study area. (*a*) Location of the study sites within the Guineo-Congolian transitional area (light green) where no changes in mean annual rainfall were recorded between 1979 and 2019 [71]. (*b*) Geological substrates as modified from Gazel *et al.* [72] underlying the Mpem and Djim national park (purple polygon) and Nachtigal (red polygon). (*c*) and (*d*) landcover change dynamics since 1975 as modified from Sagang *et al*. [15] with the age in 2020 since forest gain (green palette); forest loss (brown) and stable savanna/open vegetation (non-forest; orange). The 0.16 ha sampling plots are displayed as triangles for recent (<10 years) forests and crosses for savanna.

The study was conducted within the forest–savanna mosaics of the Guineo-Congolian transition zone [70] in the central region of Cameroon (figure 1*a*). The climate is equatorial of the Guinean type [73], with seasons alternating between dry (three dry months, mid-November to mid-March) and rainy. Average annual temperature is 25°C and mean annual rainfall is 1300 mm (between 1979 and 2019 [71]).

Savanna fires has been decreasing in the study area over the last decade (−0.75%) [74], mainly attributed to a decrease in large-scale fires (>100 ha) which are more frequent between November and February. Fire ignition in the area is mainly maintained by herders to favour the regrowth of palatable grasses for cattle. The field data collection targeted two areas, located 60 km apart, with no detected difference in MAP [75] but with contrasted paces of forest expansion after 45 years (1975–2020) of monitoring from Landsat image archives [72]. The Nachtigal area (red polygon of figure 1*b*; elevations between 450 and 550 m above sea level) lays on a metamorphic substrate and displayed a forest expansion rate of ≈0.4% year^−1^ (figure 1*d*). The proximity of the Sanaga river (blue polygon; figure 1*b*), one of the main rivers in Central Africa [76], has made the Nachtigal area an attraction for small-scale agriculture (e.g. cocoa farms, staple crops and palm plantations [77]) resulting to an increase in human pressure on the vegetation [78,79]. The Mpem and Djim National Park (MDNP, purple polygon, elevations between 540 and 650 m above sea level; figure 1*b*) mostly lies on a granitic substrate (80%, central and northeastern) where a forest encroachment rate of ≈0.63% year^−1^ (figure 1*c*) was recorded, which is slightly lower than the ≈0.66% rates recorded for its metamorphic part (20%, southern and western). Human activities in MDNP are limited by the presence of the Mpem and Djim rivers bordering the park with several rapids (blue lines in figure 1*c*) in addition to the poor quality of the infrastructures (road, bridges). However, cattle grazing occurs there during the three- month dry season.

### (b) Vegetation data

All our sampled locations were initially savanna in 1975 (figure 1*c,d*). Field campaigns took place between 2018 and 2022 (for details on the plot design, see [80]) where we established overall 73 plots of 0.16 ha (40 m × 40 m) each (59 in perpetuating savanna since 1975 and 14 in forest that transitioned in not more than 10 years). The minimum distance between plots was 100 m (median = 30 km) in savanna, and 500 m (median = 65 km) in young forests, to limit the effect of spatial autocorrelation [81,82]. The plots were distributed along a gradient of tree cover, ranging from open grassy savanna to savanna woodlands and recent forests (figure 1*c*,*d*). Forest age was determined from satellite image series [15]. Each of these plots were subdivided into four 20 m × 20 m quadrats from which the tree and grass layer was sampled. For the tree layers in the savanna plots, all woody individuals whose diameter (*D*) measured at 30 cm above the ground was at least 5 cm were identified and their diameter and total height (*H*) measured. The same census was done in forest plots for all woody individuals with a minimum *D* of 10 cm measured at breast height. A total of 2313 trees were sampled from which 76% were identified at species-level (143 woody species) and 24% at genus-level. In savanna plots, the AGB of individual trees (AGB_WOOD_, Mg ha^−1^) was computed by integrating *D*, *H* and the wood density of the individual using the allometric equation specific to savanna found in Colgan *et al*. [83]. For recent forest plots, AGB_WOOD_ was estimated by integrating the previous parameters into the pantropical allometric equation found in Chave *et al*. [84]. Wood density values were extracted from the Dryad database [85]. The BIOMASS R package [86] was used for AGB_WOOD_ estimation of individuals in forest plots. We later compiled information on the successional status for each woody species, namely savanna specialist, forest pioneers and old growth forest as referenced in local floras [87] and CoForTraits database [88].

Grass samples were collected after the growing season (November) within only 62 plots, after excluding those affected by fire at the time of sampling. For each of these plots, grass samples were clipped at the soil surface within 1 m² units placed at the centre of each 20 m × 20 m quadrat. Each fresh clipping was then oven dried to constant dry weight at 70°C. The grass layer was dominated by *Hyparrhenia diplandra*, *Hyparrhenia smithiana*, *Imperata cylindrica* and *Panicum* spp. The dry weight of the grass layer of a plot was translated to grass AGB (AGB_GRASS_) by averaging the dry weight of the clippings harvested from that plot. *Chromolaena odorata*, an invasive shrub described as favouring woody invasion of the savanna [14,20,61,89] occurred within less than 1% of our clipping units and was therefore not considered in further analyses.

### (c) Soil data

Topsoil samples (0–20 cm depth) were collected at the centre of each plot after removing litter, with the help of a handheld soil auger. The soil sample was mixed and air-dried under ambient temperature. Samples were analysed at the laboratory of the International Institute of Tropical Agriculture (www.iita.org; ISO 17025) in Yaoundé. Soil texture was determined as the proportion (%) of clay, silt and sand using the hydrometer method [90]. Soil pH in water (pH_water_) and in acid (pH_KCl_) were determined in a 1:2.5 (w/v) soil:water suspension. Soil fertility was characterized via the concentration of the main exchangeable cations (i.e. Ca^+^, Mg^+^, Na^+^ and K^+^) and cation exchange capacity (CEC) both determined using ammonium acetate method. The concentration of available phosphorus (BrayP, mg kg^−1^) was extracted using the Bray − 1 method [91]. Total P and N were determined from wet acid digestion [92] while organic carbon (C, %) was determined by chromic acid digestion [93]. Detailed analyses of soil minerals are reported by Libalah *et al*. [94].

### (d) Environmental and ecological variables

For each plot, we determined the following set of environmental descriptors: (i) fire frequency index (FFI, fire year^−1^) which quantifies the yearly rate of fire occurrence between 2014 and 2018 based on 30 m resolution Landsat imagery [15]; (ii) the elevation from the digital elevation model (DEM) [95], and (iii) distance to the nearest forest edge (distance, m) which measures the minimum Euclidean distance to the edge of the forest as mapped by Sagang *et al*. [15].

### (e) Statistical analyses

Principal component analysis (PCA) on the combination of soil and other environmental variables was used to reduce the dimensions, characterize the correlation structure and avoid collinearity issues during descriptive analysis. We focused on the key variables shaping soil texture and fertility gradients alongside fire frequency, to describe their influence on grassy and woody AGB. We used a 95th quantile nonlinear regression [96] implemented in the ‘quantreg’ package of R statistical software (v. 4.2.1) [97] to characterize the maximal effect to be expected from each of the selected variables on AGB_WOOD_ and AGB_GRASS_. Whenever applicable, we identified the breakpoints at which maximum AGB_GRASS_ was attained using ‘segmented’ package in R. We used a 50th quantile linear regression to describe the median effect on AGB_WOOD_ and AGB_GRASS_ of the selected variables in presence of possible effects from other variables, and tested whether the regression slopes were different from zero. Subsequently, significant differences between localities or geological substrates were tested using post-hoc paired comparisons from the Student’s *t*‐test. The spatial dynamics of woody savanna specialists and forest pioneers across a gradient of forest–savanna transition was indirectly assessed using plots data to quantify their contribution to species abundance and AGB_WOOD_ within successive 20 m bins, starting from the forest edge and extending towards the interior of each ecosystem (up to 100 m). The main floristic patterns in woody species composition were identified by applying a non-symmetric correspondence analysis (NSCA) [98,99] to the plot (*n* = 73) × woody species (*n* = 143) cross-classification table. NSCA analyses the species abundance (number of individuals) in plots to identify compositional gradients by putting emphasis on abundant species and is thus robust against casual correspondences between plots with few individuals and rare species [100]. We focused on species’ scores along the most dominant axes of the NSCA to establish the gradients in woody species composition. We used the envfit function from the vegan R-package [101] to evaluate the linear correlation between the first two axes of the NSCA and soil and the key additional environmental variables.

## 3. Results

The PCA (electronic supplementary material, appendix S1) summarizing the correlations between soil properties and other environmental variables revealed two main gradients (58% of total variance explained; electronic supplementary material, appendix S2a). The first gradient (PCA 1 with 35% of variance explained) was driven by soil fertility variables (negative correlation with exchangeable cations, pH and silt content) and distinguished soils found on granite (mean scores on PCA 1 = 2.15; s.d. = 1) from those found on metamorphic substrate (mean scores on PCA 1 = −1.12; s.d. = 2.5). We further retained silt content and CEC as our descriptors of soil fertility considering that both parameters have significant contribution to PCA 1 and show loose intercorrelation. PCA 2 (23% of variance explained) was driven by soil textural variable and contrasted sandy versus clayey soil conditions. Even though the highest fractions of clay were found on granite in the MDNP, its southwestern part displayed metamorphic substrate and less-clayey soils, so that average PCA 2 scores of plot scores did not differ between both localities based on a post-hoc paired comparisons from the Student’s *t*‐test (electronic supplementary material, appendix S2; *p*‐value ≤ 0.05).

### (a) Effects of soil on wood and grass–biomass interactions modulated by fire

**Figure 2.**
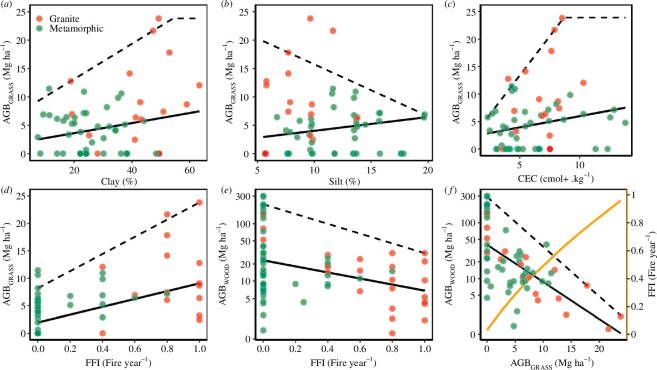
Relationships between soil properties, FFI and AGB of grassy (AGB_GRASS_) and woody (AGB_WOOD_) layers. (*a–d*) Influence of soil texture (clay and silt content), fertility (CEC) and FFI on AGB_GRASS_. (*e*, *f*) Influence of FFI on the woody layer with its effects on the relationship between AGB_WOOD_ and AGB_GRASS_. Colours denote plots located on granite (red) and metamorphic (green) substrates. Broken and unbroken lines, respectively, represent the 95th and 50th nonlinear piece-wise quantile regressions. The unbroken orange curve in (*f*) shows the mean FFI along the gradient of AGB_GRASS_.

The median trend (represented by solid black line in figure 2*a-c*) indicated an increase in AGB_GRASS_ with higher soil texture and fertility, although the slopes were not significantly different from zero. Soil clay content drives the upper bound (represented by solid dotted line) of AGB_GRASS_ (figure 2*a*); with highest values (>12 Mg ha^−1^) only reached on granitic substrates supporting clay-rich soils, and a peak found at very high clay levels (>40%). Conversely, AGB_GRASS_ showed an overall decrease of its upper bound towards higher silt contents (figure 2*b*) and peaked at intermediate levels of CEC (between 5 and 10 cmol + kg^−1^; figure 2*c*).

We noticed a significant increase in the median and upper bound on AGB_GRASS_ with increasing FFI (figure 2*d*). All plots exhibiting low FFI (<0.4 fire year^−1^) displayed lower AGB_GRASS_ (<12 Mg ha^−1^). Conversely, the highest values of AGB_GRASS_ (approaching 25 Mg ha^−1^) were recorded under FFI exceeding 0.6 fire year^−1^, where only two plots had AGB_GRASS_ below 5 Mg ha^−1^. AGB_WOOD_ decreased with FFI and all plots displaying non-null FFI had AGB_WOOD_ values below 40 Mg ha^−1^ (figure 2*e*). Plots with no fire detection (FFI = 0) showed a wide range of AGB_WOOD_ irrespective of the geological stratum. There was a general decreasing relationship between AGB_WOOD_ and AGB_GRASS_ (figure 2*f*), the latter limiting AGB_WOOD_ below 40 Mg ha^−1^ once exceeding 5 Mg ha^−1^ of AGB_GRASS_.

### (b) Patterns in woody species composition and structure along a forest–savanna gradient

**Figure 3.**
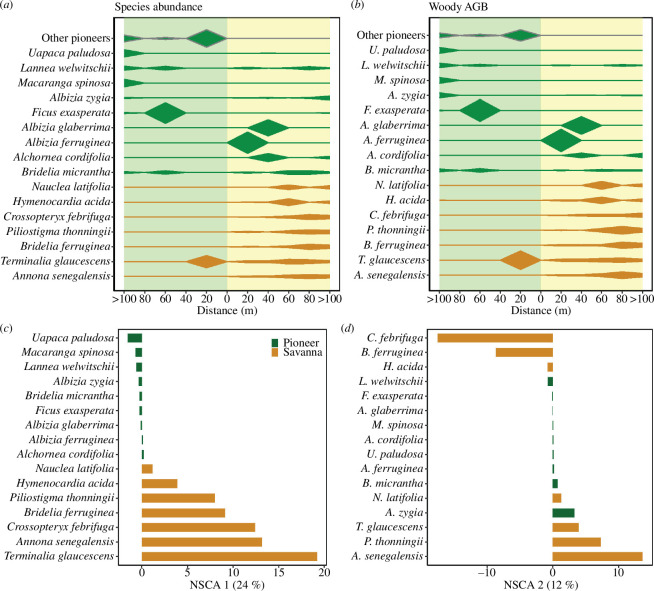
Patterns in species composition and structure of the woody layer. (*a,b*) Contribution in species abundance and AGB_WOOD_ of woody species based on their distance from the forest–savanna boundary (distance = 0) towards the young forest (light green background) or savanna (yellow background). The polygons represent the variation in (*a*) relative abundance and (*b*) AGB_WOOD_ of savanna specialists (orange polygons) and forest pioneers (dark green polygons) from plots grouped within successive distance bins of 20 m. The size of polygons is proportional to the contribution of each species. (*c–e*) Species ordination along the first two axis from a NSCA. The proportion of total variance explained by each axis is given in parentheses. Orange and green bars are species scores, respectively, for savanna specialists and forest pioneers with the highest contribution to the axis.

When tracking the contribution of woody species to the abundance and biomass across a forest−savanna mosaic, we notice the prevalence of forest pioneers including *Alchornea cordifolia*, *Albizia ferruginea* and *Albizia glaberrima* within plots in the first 20–60 m, as we move from the forest edge (distance to plot = 0) into the savanna (yellow background in figure 3). It should be noted that no flammable AGB_GRASS_ was observed in plots located within this distance range. *Terminalia glaucescens* was the only savanna specialist which together with forest pioneers showed noticeable contribution in the species abundance and AGB_WOOD_ within the first 40 m into the forest. Beyond ~60 m from both sides of the forest–savanna boundary, species composition was more specific to one of these two physiognomies except for forest pioneers *Albizia zygia*, *A. cordifolia*, *Bridelia micranta* and *Lannea welwitschii* whose abundances were still noticeable within savanna.

The NSCA highlighted two dominant gradients of woody species composition (figure 3*c–d*) which together explained 39% of total variance. The first axis (NSCA 1, 24% of total variance) opposed savanna (positive end) with forest specialists (negative end, dominated among others by *Uapaca paludosa*, *Macaranga spinosa* and *L. welwitschii*) and reflects a gradient of floristic transition as forest encroaches over savanna.

The second axis (NSCA 2; 15% of total variance), highlighted two contrasted communities of savanna species. *Annona senegalensis*, *Piliostigma thonningii* and *T. glaucescens* dominated the positive end of NSCA 2, with a noticeable contribution of a forest pioneer *A. zygia*. The negative end was driven by *Bridelia ferruginea*, *Crossopteryx febrifuga* and *Hymenocardia acida* (figure 3*d*).

### (c) Relationship between soil and other environmental factors with woody structure and species composition

**Figure 4.**
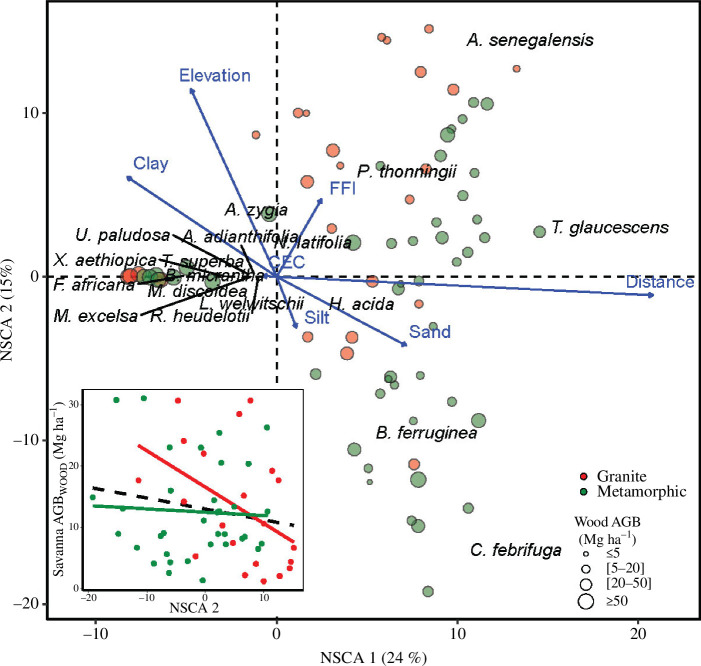
Correlations between soil and other environmental variables over plots and the first and second axis of NSCA of woody species. Blue arrows represent the projection of soil and other environmental variables, with their lengths proportional to the correlation with the axes. Circles represent plots found on granite (red) and metamorphic (green) substrates with their sizes proportional to AGB_WOOD_. The inset shows linear regressions between AGB_WOOD_ and NSCA 2 only for savanna plots (dotted line) over each geological substrate (granite = solid red, metamorphic = solid green).

Variables related to soil texture exhibited stronger correlations with NSCA 1 as compared with soil fertility variables (figure 4). NSCA 1 showed a strong positive correlation with the distance to the forest edge (*r* = 0.6). NSCA 1 was also negatively correlated to clay content (*r* = −0.3) even though the clayey versus sandy plots gradient went obliquely to the NSCA axes. Clayey plots were either near the negative end of NSCA1 (dominated by forest pioneers) or not far away from the centre of the axis), while sandy sites displayed a dominance of savanna specialists (positive end). Nearly, all plots with AGB_WOOD _>40 Mg ha^−1^ had negative scores along NSCA 1 and were dominated by forest pioneers.

The relationship between NSCA 2 and soil and environmental variables was more complex. NSCA 2 was positively correlated with elevation, clay content and FFI. Plots with high values of FFI showed a dominance of *A. senegalensis* and *P. thonningii*. Conversely, silt and sandy sites which showed negative correlation with NSCA 2 were dominated by *B. ferruginea* and *C. febrifuga*. When focusing on savanna vegetation, the dichotomy observed in species composition is also reflected in the structure, with AGB_WOOD_ showing a decreasing relationship with NSCA 2 (solid dotted line in the inset of figure 4). This decrease was more pronounced and significant on granite (*r* = −0.5) as compared with metamorphic substrate (*r* = −0.1).

## 4. Discussion

The expansion of forest over savanna is of continental [12] and global significance [1]. This calls for a better understanding of the underlying modalities and mechanisms that drive tree–grass coexistence across diverse environmental conditions. Here, we analysed collocated data about grass, trees and fires in the humid part of the savanna biome. With respect to the questions posed at end of §1, we found (i) a strong influence of soil texture on grass production and fire frequency. (ii) Woody AGB remained low in areas with strong grass–fire feedback, while increasing woody AGB strengthened the tree shading effects on grasses. (iii) The structure and composition of the woody layer within the forest–savanna ecotone confirmed a rapid extension of the forest edge, with (iv) a strong influence of the distance to the forest boundary and soil texture.

### (a) Soil modulation of the fire-mediated tree–grass interactions

A central, well-established concept in savanna ecology is the grass–fire feedback: high grass production during the rainy season favours both fire spatial propagation and severity during dry months [34,102,103]. Here, AGB_GRASS_ increased with clay-content and reached its highest (up to 24 Mg ha^−1^) on granitic substrate displaying intermediate levels of soil fertility (CEC ranging between 5 and 10 cmol + kg^−1^). This appears consistent with statistical results indicating that grass biomass increases more directly with increasing clay content than it does in response to increasing soil nutrient availability [46]. Increasing AGB_GRASS_ favours higher fire frequencies and intensity [57,104], with effects on woody plants that vary among species though always depressing the overall woody biomass. We document here evidence of a sharp nonlinear increase in observed FFI (based on Landsat) beyond a pivotal range of AGB_GRASS_ between 3 and 5 Mg ha^−1^. AGB_GRASS_ below 4 Mg ha^−1^ was mentioned as limiting for fire propagation and/or effectiveness in several situations within the savanna biome [36,37,55,105,106]. A lower yet similar threshold of 2.5 Mg ha^−1^ was observed as the minimal value to ensure fuel connectivity and fire spread from a more extensive study [30]. However, during field sampling, small-extent fires with low intensities, or fire scars on tree bark, could be observed within plots where AGB_GRASS_ was below 4 Mg ha^−1^. These small-scale fire events were probably missed from remote sensing products due to limitations in both spatial and temporal resolution of Landsat image series [107]. Consequently, very low fire frequencies (FFI close to 0) were recorded for these areas. Beyond an AGB_GRASS_ threshold of 4 Mg ha^−1^, FFI escalated to over *ca* 0.8 year^−1^ suggesting a burning regime approaching large scale annual fires [74]. The FFI proxy thus allowed us to sort out two kinds of scenarios: one characterized by large variance in AGB_WOOD_ and low AGB_GRASS_, where fires were either absent or too small to be detected with Landsat-based FFI (FFI = 0), and a second scenario with non-null FFI values where AGB_WOOD_ is consistently low (<40 Mg ha^−1^; figure 3*b*) with higher AGB_GRASS_ (>5 Mg ha^−1^). Overall, we noticed a depressing effect of FFI on AGB_WOOD_ from plot data (figure 2*e*). This finding aligns with the results from a broader survey in the area covering approximately 300 ha using satellite and air-borne Lidar data (see [15], and additional specific results in electronic supplementary material, appendix S3), which described a decreasing pattern of low AGB_WOOD_ (<40 Mg ha^−1^ and median values of *ca* 20 Mg ha^−1^) for FFI > 0.6 fire year^−1^. FFI exceeding 0.4 year^−1^ systematically corresponded to low AGB_WOOD_ (<40 Mg ha^−1^). This leaned towards the widespread idea that frequent fires of large extent, fostered by high grass standing crop can maintain AGB_WOOD_ at very low levels in humid savannas [36,55,108], thereby keeping the grass–fire feedback effective. Conversely, situations associating intermediate fire frequency (0–0.4 fire year^−1^) and AGB_WOOD_ values above 40 Mg ha^−1^ are barely observable in the studied savannas (electronic supplementary material, appendix S3). This is likely a consequence of rapid savanna transition towards close canopy forests in those areas, due to the strength of the tree–shade depressing feedback. In both cases, necessary conditions for hysteresis loops are met.

Our results from plot data confirmed the suppressing effect of AGB_WOOD_ on AGB_GRASS_ through leaf shading and/or root competition [109,110], since all our sampled situations with AGB_WOOD_ above 40 Mg ha^−1^ corresponded to very low AGB_GRASS_ (<4 Mg ha^−1^, figure 4*c*). The AGB_WOOD_ versus AGB_GRASS_ decreasing relationship appeared clearer in the most productive environments (as referenced by AGB_GRASS_ > 10 Mg ha^−1^), mainly occurring on clayey soils. Thus, the two opposing positive feedback (grass–fire versus tree–shade), each prevail according to identified thresholds in grassy and woody state variables and currently prevail in distinct areas of our study site. The simultaneous occurrence of forests and very open grassy savannas is particularly striking on clayey soils and compatible with an interpretation of AES (yet of undefined levels of stability). The ecotone at the forest edge is a zone of sheer opposition between these two positive feedback loops, where vegetation may react to changes or variation in external forcings in the most direct and dramatic way.

### (b) Structure and composition of the woody layer across a forest–savanna boundary

Over the last five decades, the study area experienced a massive encroachment of forests over savanna [15], and all our sampled locations (both savanna and recent forests plots) were initially savanna in 1975 (figure 1). We observed that the two tallest among frequent savanna species (i.e. *T. glaucescens* and *H. acida*) not only occurred on the savanna side at all distances to the edge but also had shares of AGB_WOOD_ peaking in the most recent forests, closer to the forest boundary. Those two species neither regenerate nor survive for long in forest environments, and the observed individuals are of tall stature and have been overtopped by forest pioneers quite recently (because they were not found farther than 40 m from current edges). This is indicative not only of the past savanna state (also known from satellite series) but also of the favourable growth conditions close to forest edges as their AGB_WOOD_ shares were higher than in neighbouring savanna. In these situations, a large portion of AGB_WOOD_ was also accounted for by some forest pioneers (such as *Albizia* spp. and *Alchornea* spp.), which have already been reported as showing a certain degree of fire-tolerance and contributing to forest boundary movement within the forest–savanna ecotone [14,20,59,61,111]. This species succession was further reflected in the main floristic gradient (NSCA 1, figure 3) opposing forest pioneers with savanna specialists, which was correlated to sand/clay content unlike soil fertility. Forests pioneers were more dominant on clay-rich soils as soon as lower fire frequencies prevailed (indicating AGB_GRASS_ depression by woody cover). This contrasts with sandy/silty soils where savanna community was of low woody AGB despite lower fire regimes (figure 4). This leads to sharper boundaries on clay soils and a more specialized composition of species than reported in some other sites experiencing similar climate and rainfall (MAP of 1300–1400 mm, dry season of 3–4 months). Notably, in Ghana, a set of ‘non-specialized’ woody species identified along a forest–savanna gradient on soils which were heavily sandy (>84%) and nutrient poor (CEC < 7.5 cmol dm^−3^) [63], led to the conclusion that a distinctive floristic and tree canopy cover gradients was absent in the forest savanna ecotone. Although AGB_GRASS_ is not available in Ametsitsi *et al*. [63], it is likely to be quite low, with limited ‘fire sharpening’ effects on the floristic composition. We thus delineated two distinct situations of woody savanna structure and species composition that played a pivotal role in shaping the ecological dynamics of the ecotone.

### (c) Implications for landscape-scale dynamics of the forest–savanna ecotone

The central and northeastern MDNP which is found on granite substate (dominated by clayey soils) displayed the highest range of grass production along with high fire frequency (mean FFI ~0.5 fire year^−1^ at plots scale). This is, however, concomitant to rapid afforestation (0.63% year^−1^ with respect to total area), principally due to the pervasive forest boundary expansion (figure 1). Although the overall fire regime seemed able to keep AGB_WOOD_ under the threshold of 40 Mg ha^−1^ in the middle of large savanna patches, there were local fire-resistant/-attenuated situations, such as those close to forest boundaries (as already reported from Lopé, Gabon [13]), near small nucleation groves (as reported in littoral Congo [16]), or in savanna patches surrounded by forests [58,59]. Fire regimes in the MDNP are mainly maintained by herders who burn to promote palatable grasses for cattle. Fire ignition is, however, insufficient in some areas, which are often remote from core grazing areas and become rapidly colonized by forbs, vines and pioneer forest species as in Youta-Happi *et al*., Achoundong *et al*., Fandohan *et al*. and Deklerck *et al.* [14,61,89,112]. The southern and western MDNP found on metamorphic substrate (dominated by coarser soil texture) displayed lower Landsat-detected fires frequencies (mean FFI ~0.3 fire year^−1^ at plots scale over the stable savanna) and a slightly higher pace of forest encroachment (0.66% year^−1^). Forest expansion in this area mainly occurred along boundaries of preexisting forests (figure 1). We interpret it as consequence of loose fire ignition practices in a context of lesser fire propagation (due to lower AGB_GRASS_ recorded on metamorphic substrate). Lastly, in the Nachtigal area where herding activities are coexist with agricultural practices [77], we noticed the slowest pace of afforestation (0.4%) [15], and an AGB_GRASS_ similar to southern MDNP and FFI close to zero. Field inspections, however, confirmed that fire occurred in most of the areas, although most of them are too small to be detected by the Landsat FFI proxy. Our analysis presents high similarities in soils, AGB_GRASS_/AGB_WOOD_, and floristic woody composition between Nachtigal and southern MDNP, which suggests that contrasting observed encroachment rates result from differences in human practices. Forest edge expansion in Nachtigal could be hampered by an anthropogenic edge effect [69] involving casual tree removal or wood cutting in savanna close to agricultural areas (i.e. cocoa plantations, fallows). Moreover, in very similar humid savannas of Congo, it has been highlighted that subtle variation in human activities and fire management can either speed-up or slow-down the overall forest expansion trend [113].

## 5. Conclusion

Our results captured and quantified the positive feedback loop involving grass production, fire propagation and control of woody species composition in a context of humid savanna transitions and ample soil texture and fertility gradients. We here show how soil texture modulates the grass–fire feedback by allowing stronger grass production and easier fire propagation on clayey soils compared with sandy textures. We also provide quantitative thresholds about the negative feedback of woody biomass on grass biomass, a process which is scarcely documented in literature, and we highlight a threshold of *ca* 40 Mg ha^−1^ of woody biomass above which the grass–fire feedback loose effectiveness and vegetation rapidly transition to forest. Clayey soils displayed the most contrasted vegetation mosaics (grassy savannas versus recent forests), which can be seen as AES. However, the long-term maintenance of grassy savannas by the grass–fire hysteresis loop is only granted far from forest edges. This suggests that currently observable mosaics are transitory under the present conditions (climate, CO_2_ concentration, dominant anthropogenic fire regimes) since swift forest boundary movement was observed over the last four decades. Forest edges were sharp and floristically well characterized by advancing forest pioneers (e.g. *Albizzia* spp.) and overtopped savanna trees, yet were well developed (e.g. *T. glaucescent*, *H. acida*) and did not suggest any important role of generalist species liable to perpetuate in both savanna and forest contexts. At present, the largest share of existing literature about humid savannas has addressed sandy oligotrophic situations. Orienting further field studies and meta-analyses so as to thoroughly quantify vegetation properties and dynamics, while sampling the entire soil texture and fertility gradients in the forest–savanna transition zone, would provide a sound basis for modelling and anticipating the future of the extensive forest–savanna ecotone in the face of global change.

## Data Availability

The dataset and codes used in this study are permanently accessible in this repository [114]. Supplementary material is available online [115].
